# Enhanced imaging in low dose electron microscopy using electron counting

**DOI:** 10.1016/j.ultramic.2009.07.004

**Published:** 2009-11

**Authors:** G. McMullan, A.T. Clark, R. Turchetta, A.R. Faruqi

**Affiliations:** a MRC Laboratory of Molecular Biology, Hills Road, Cambridge CB2 0QH, UK; b STFC Rutherford Appleton Laboratory, Chilton, Didcot OX11 0QX, UK

**Keywords:** DQE, MTF, CMOS, Electron counting

## Abstract

We compare the direct electron imaging performance at 120 keV of a monolithic active pixel sensor (MAPS) operated in a conventional integrating mode with the performance obtained when operated in a single event counting mode. For the combination of sensor and incident electron energy used here, we propose a heuristic approach with which to process the single event images in which each event is renormalised to have an integrated weight of unity. Using this approach we find enhancements in the Nyquist frequency modulation transfer function (MTF) and detective quantum efficiency (DQE) over the corresponding integrating mode values by factors of 8 and 3, respectively.

## 1 Introduction

The signal to noise ratio in an electron microscope image of a radiation sensitive sample is limited by the small number of incident electrons that can be used to form the image before radiation damage destroys the specimen. In the study of radiation sensitive samples the electron detector performance therefore assumes a greater importance than with less radiation sensitive samples. A convenient way to measure the performance of a detector is with the detective quantum efficiency (DQE). At zero spatial frequency this is defined as

(1) $$\mathrm{DQE}={\left(S/N\right)}_{\mathrm{out}}^{2}/{\left(S/N\right)}_{\mathrm{in}}^{2}$$

in which (S/N) indicates the signal to noise ratio. An ideal detector faithfully records all incident electrons with the same response and without introducing additional noise. Such a detector has a DQE of unity but all real detectors have lower values. For imaging applications the generalisation of Eq. (1) to finite spatial frequency, ω, is important. This can be expressed as [1–3]

(2) $$\mathrm{DQE}(\omega )=d_{n}^{2}\mathrm{MTF}{\left(\omega \right)}^{2}/n{\mathrm{NPS}}_{n}(\omega )$$

in which MTF(ω) is the modulation transfer function, *n* is the number of incident electrons per pixel, $d_{n}$ the average output signal, ${\mathrm{NPS}}_{n}(\omega )$ the noise power spectrum of the image and the output signal per incident electron is given by $d_{n}/n$. The subscript *n* on $d_{n}$ and ${\mathrm{NPS}}_{n}(\omega )$ is used to emphasise that they are the values obtained with *n* electrons per pixel. From Eq. (2) it can be seen that a high MTF(ω) is advantageous in achieving a high DQE(ω) but not essential provided there is a corresponding drop in the ${\mathrm{NPS}}_{n}(\omega )$.

Monolithic active pixel sensors, MAPS, show great promise as direct detectors of electrons with energies between 100 and 300 keV typically used in an electron microscope [4–8]. They combine the advantages of film (which result from detecting incident electrons in a thin, i.e. ~10μm, sensitive layer) with the low noise, fast readout, and immediate feedback of electronic detectors. They are fabricated using standard CMOS technology which enables them to be produced with large numbers of pixels, e.g., 4k×4k. The exposure to high energy electrons in direct detection does however lead to radiation damage in the detector itself. The initial consequence of this is an increase in leakage current but by using standard radiation hardening (radhard) design techniques, along with fast readout and cooling, it is possible to make a detector that is usable even after an exposure to more than 1 MRad [9,7]. While insufficient for many applications, this level of radiation hardness is sufficient for several years of careful use in low dose applications such as cryoEM or cryo-tomography.

In a MAPS detector, incident electrons or photons are detected via the voltage drop across a capacitor resulting from the collection of mobile charge associated with electron–hole pair excitations generated in a sensitive layer consisting of a highly ordered epitaxially grown semiconductor. To maximise the response and minimise the reset noise, the associated capacitance is usually kept as small as possible [10]. In the detection of visible light the number of electron–hole excitations generated per incident photon and corresponding voltage drop per photon, is small. The detector is therefore operated in what we will call an integrating mode, in which the voltage drop is measured after a fixed time and contains the sum of contributions from a large number of incident photons. MAPS detectors recording incident electrons are conventionally also operated in this mode. The number of electron–hole pairs generated per incident electron is however much larger and to avoid saturation the number of electrons incident during the integration time must be kept low. The actual numbers are design dependent but at 120 keV the average number of incident electrons per pixel in an individual frame typically has to be kept below 10. As a result even a typical low dose image, with just 25 electrons per pixel, has to be accumulated over several frames. The sensitivity of MAPS detectors to incident electrons does however allow the detection of individual incident electrons. This makes it possible to generate an image using what we will call the single electron event mode, in which the final image is assembled from the information contained in the images of the individual incident electrons. In order to do this, it must be possible to distinguish the individual events and so the total number of electrons incident per frame must be kept very low. As a result, a low dose image collected in single electron event mode has to be generated from several thousand individual frames.

The performance of a MAPS detector in recording electrons can be optimised through the choice of pixel size as well as the thickness of the passivation, sensitive layer and substrate together with the use of low noise readout. When operated in integrating mode the performance is limited by a number of factors. In particular the intrinsic variability of the energy deposited along the stochastic trajectory followed by an incident electron through the sensitive layer and the blurring of the resulting signal by the diffusion of charge carriers within the sensitive layer. In this paper we show that it is possible to minimise these factors and produce improved images by computational analysis of the signals from individual incident electrons.

## 2 Methods

Images were recorded on an experimental MAPS detector fabricated as part of the MI3 collaboration [11]. The detector is known as a Vanilla detector and has a non-radhard three transistor (3T) pixel design [12,10]. Each pixel has an area of $25\times 25\;\mu m^{2}$ and the whole detector consists of 520×520 pixels. The detector is read out using a rolling shutter via 12-bit successive approximation ADCs fabricated on the sensor. There is one ADC for every four columns and with these the whole sensor can be readout at a maximum rate of 148 fps. The operation of the sensor is controlled by custom FPGA software that allows it to be reset and readout in different modes and at different speeds. The results presented here were obtained using a hard reset [10] and the sensor integration time, and corresponding frame rate, adjusted using time delays at the end of each frame. Output from the sensor is transferred via gigabit Ethernet for processing and storage on a dedicated computer.

The detector was mounted at the plate camera position in a Philips CM12 microscope operating at 120 keV. The electron dose was calculated from the microscope exposure meter which was cross calibrated, via film images, with a Faraday cup on an adjacent microscope. The actual number of electrons per frame was controlled by varying the magnification of the microscope. Single event images were typically collected using approximately 1 electron per 500 pixels so that a data set with a total of 15 electrons per pixel requires 55 s to collect, consists of nearly 8000 frames and requires 4 GB of storage. While it is useful to retain all the images it was also possible to retain only the single events or simply the final processed image. The detector does not employ correlated double sampling and all images are dark subtracted and gain normalised.

To find the single events we first search for a seed pixel in which the signal is above a fixed threshold relative to the background. Having found a seed pixel the remainder of the corresponding event is obtained by including all neighbouring pixels that either have signal above the threshold, or are within a given distance of any other pixel that is above the threshold. Because of the high signal to noise, the choice of threshold is not critical. The threshold is typically taken to be as low as possible, so as to minimise the number of true events missed, but yet sufficiently high, so as to avoid generating false events. As the size of each event is limited, the search can be carried out in a cache efficient manner along a row following a few rows behind the current rolling shutter readout window. This enables the finding and processing of the individual events to be carried out during the acquisition of images, though in practice the analysis presented here was done off-line.

Single event images can be analysed in many ways. Simply counting those pixels in which the signal exceeds a given threshold mimics the behaviour of the Medipix2 hybrid pixel detector [13,14]. A problem with this procedure is that individual events can be recorded in more than one pixel. This introduces a variability in response to an incident electrons that results in a decreased DQE. To avoid this, individual events need to be identified and given the same weight, no matter how many pixels they are spread over. One way to do this is to choose one pixel to represent the event. This could be the pixel with the largest signal or the pixel containing the weighted centroid of the event. Assigning the full weight of the incident electron to this pixel removes the intrinsic variability in response to an incident electron and leads to an improved low spatial frequency DQE(ω). However, restricting each event to a single pixel removes the fall in the ${\mathrm{NPS}}_{n}(\omega )$ with increasing ω and leads to DQE(ω) being proportional to $\mathrm{MTF}{\left(\omega \right)}^{2}$ (see Eq. (2)). With this method it is important that the MTF is as high as possible and so ensure that a chosen pixel accurately represents the arrival position of the incident electron. Ideally one would like to be able to infer from an event the position at which the electron was incident on the detector. If this can be done with sub-pixel resolution it would be possible to overcome the sin(x)/x pixel damping envelope which otherwise restricts the MTF at the Nyquist frequency to 2/π. From the observed single events, Bayesian inference can be used to generate a probability distribution for the arrival position of the electrons. Unfortunately, the diffusion blurred images obtained with the Vanilla detector's $25\times 25\;\mu m^{2}$ pixels do not in general retain sufficient information about the energy loss along the stochastic trajectory of an incident 120 keV electron in order to accurately identify the electron's arrival position. Analysis of simulated events in which calculated electron trajectories and measured charge diffusion effects are used as priors, does however indicate that the signal in a pixel approximately reflects the calculated probability distribution obtained using a Bayesian analysis. Based on this observation, our preferred way of processing the single event images is a heuristic approach in which we ignore any contributions below the threshold for the event and renormalise the remaining contributions so that they sum to unity. The resulting effective probability distribution is fast to calculate and yet captures the main features of the more computationally expensive Bayesian analysis. With this approach all events contribute equally to the ${\mathrm{NPS}}_{n}(\omega )$ at zero spatial but events that are spread over several pixels contribute less at higher spatial frequency.

The MTF was measured using the well-known edge method [1] with a 2 mm diameter Pt rod placed at the pointer position of the microscope being used to create the edge. The processed images were analysed using the same approach and computer programs used in [8]. In particular the edge spread function was fitted to a model for the point spread function consisting of a sum of up to three Gaussian functions.

The DQE(ω) of the detector in integrating mode was calculated via Eq. (2) using the measured number of electrons per frame, ${\mathrm{NPS}}_{n}(\omega )$ calculated from the power spectra of the difference between successive frames and previously calculated MTF(ω). When operated in single event mode, the DQE(ω) was obtained from an estimate of DQE(0), the measured MTF(ω) and the ${\mathrm{NPS}}_{n}(\omega )$ obtained from the circular average of the sum of power spectra of the individual renormalised events [2]. DQE(0) is obtained from the ratio of the number of recorded events to the expected number of incident electrons.

## 3 Results

**Fig. 1:**
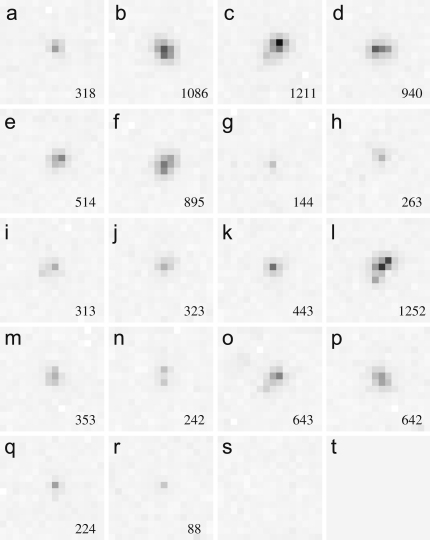
Examples of single electron events due to 120 keV incident electrons as recorded on the Vanilla sensor. The pixel values are both dark subtracted and gain corrected. Their values lie between -15 and 241 ADC units and they are illustrated by linearly mapping their values to a grey scale in which the incident electrons are shown as darker. A typical area with just readout noise is shown in (s) and, for reference, the zero value is illustrated in (t). The integrated value of each event is given in the lower right-hand corner of each panel. The average integrated value for 120 keV electrons was found to be 375 ADC units. In the images shown the peak value in an individual pixel is 241 ADC units and is found in event (c). The peak value in the weakest event shown in (r) is 49 ADC units.

In Fig. 1, panels (a)–(r) show examples of typical single electron event images obtained at 120 keV. For comparison the noise level is illustrated with an area having no events in panel (s) and the zero level is shown in panel (t). The reason for wanting to use the electron event counting mode instead of integrating mode is illustrated by the difference in the way the events in Fig. 1(b) and (r) are treated. The event shown in Fig. 1b contains 12× the integrated signal of that in Fig. 1(r) but it is the latter event that contains the high spatial frequency information and it is weak precisely because it comes from an incident electron that passes relatively unscattered through the sensitive layer. In the integrating mode the low resolution event ends up with greater weight whereas in the single electron event mode the weight of the high resolution event is increased and both events correctly have the same weight at zero spatial frequency.

**Fig. 2:**
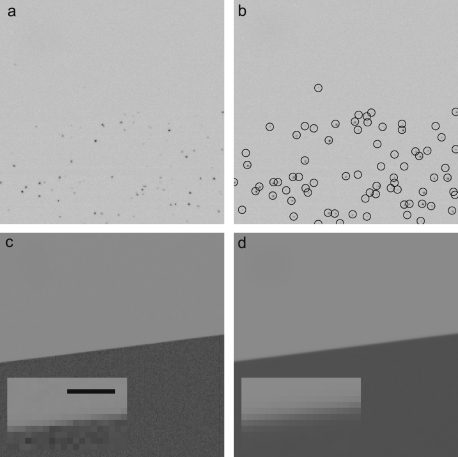
Images of a 300×300 pixel area of the detector illuminated by the edge shadow pattern used to calculate the MTF. The pattern is formed by partially blocking a uniform beam with a 2 mm diameter Pt rod placed in the pointer position of the microscope. (a) Shows a typical frame in a series used to calculate the edge image via single electron events. As in Fig. 1, darker areas indicate the single electron events. (b) Shows the events selected from the frame shown in (a). The circles have been drawn around the seed pixel associated with each event. The use of a threshold meant that some events are not counted and where two incident electrons are too close they are counted as a single event. (c) Shows the edge image obtained by summing the contributions from single electron events contained in 68 550 frames such as shown in (a). The events have been renormalised using the procedure described in the text. (d) Shows the corresponding edge image obtained using the MAPS detector in the integrating mode in which the individual dark subtracted and gain normalised frames are simply added. The insets in (c) and (d) show an 8× magnified view of an area of the edge with a scale bar representing 8 pixels, i.e. 200μm.

Fig. 2 shows a 300×300 pixel area on the detector used in the measurement of the MTF via the edge method. Fig. 2(a) shows a typical frame in the series used to calculate the MTF using single electron events. In Fig. 2(b) the selected events from the image shown in Fig. 2(a) are marked with a circle centred on the seed pixel. Fig. 2(c) and (d) show the final edge images obtained from the summation of all frames using the single electron and integrating modes, respectively. The insets in Fig. 2(c) and (d) contain 8× magnified areas around the edge and clearly illustrate the improved MTF obtained using the single event mode.

**Fig. 3:**
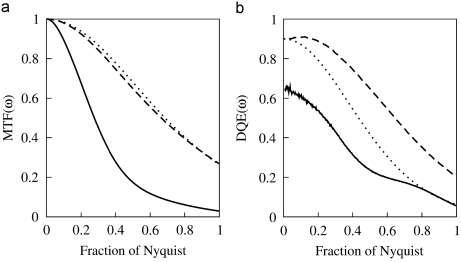
Comparison of (a) MTF (ω) and (b) DQE (ω) obtained using integrating and single electron event modes. Results obtained using the integrating mode are shown as the solid (—) lines. Single electron mode results in which renormalised events are used are shown in dashed (-) lines and single electron mode results obtained using the events’ peak positions are shown as dotted (⋯) lines.

A comparison of the measured MTF(ω) and DQE(ω) results obtained using the integrating and single electron counting modes of the Vanilla detector is given in Fig. 3. Single event image results obtained using the peak pixel position of an event and the heuristic event renormalisation procedure introduced in Section 2 are given. The integrating mode MTF was obtained with a three Gaussian fit and falls to 3% at the Nyquist frequency. A double Gaussian fit was sufficient to describe the single event MTF and the results obtained using either the peak position or renormalised event distributions are almost identical. In both cases the MTF at the Nyquist frequency is 25% and represents an eight-fold improvement over the corresponding integrating mode result. The value of DQE(0) in the integrating mode was independently measured via the binning method described in [8] to be 65%. This agrees well with the zero frequency limit of DQE(ω) shown in Fig. 3b. The corresponding value at the Nyquist frequency is 6%. The value of DQE(0) in the single event mode was estimated to be 90% based on the ratio of counted to expected number of incident electrons. When the peak position of events is used to analyse the single event images the resulting DQE(ω) falls as $\mathrm{MTF}{\left(\omega \right)}^{2}$ with increasing ω. Despite the 8× enhancement of the MTF, there is no difference in the value of DQE at the Nyquist frequency between that calculated using the single event peak position and that obtained using the integrating mode. In contrast, using the renormalised event method to analyse the single electron event images produces an enhanced DQE(ω) at all spatial frequencies. In particular the value of 20% at the Nyquist frequency represents a three-fold improvement over both the corresponding integrating and single electron peak position results.

**Fig. 4:**
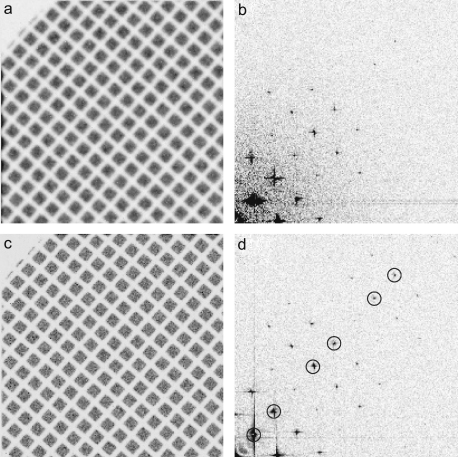
Comparison of integrating and single electron mode shadow images of an EM grid obtained using the same total number of electrons. The integrating mode and single electron mode images are shown in (a) and (c), respectively, with the darker areas representing areas exposed to electrons. A quadrant of the Fourier transforms of the images shown in (a) and (c) are given in (b) and (d), respectively. The circles in (d) indicate the spots from one of the [10] directions whose amplitudes and signal to noise ratios were measured and used in generating Fig. 5.

**Fig. 5:**
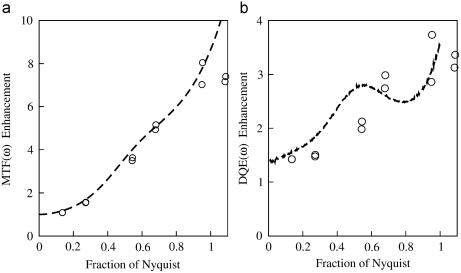
Enhancement in (a) MTF (ω) and (b) DQE (ω) performance obtained by using renormalised single event imaging over the conventional integrating mode imaging. The dashed lines in (a) and (b) show the corresponding enhancements taken from the results given in Fig. 3. The circles (∘) show results obtained from the grid shadow images. The MTF enhancement was obtained from the ratio of amplitudes of the spots as indicated in Fig. 4 measured in single event and integrating modes. The DQE enhancement was obtained from the corresponding square of the ratio of signal-to-noise for the spots.

The enhanced imaging performance possible by using the single electron mode is further illustrated in Fig. 4 using shadow images of an EM grid. The grid was mounted at the pointer position of the microscope and shadow images were taken with a dose of 15 electrons per pixel. The integrating mode image shown in Fig. 4(a) came from 5 frames and was acquired in 0.1 s while the corresponding single electron image shown in Fig. 4(c) was obtained using renormalised events from 7500 frames and took 51 s to acquire. The single event image appears sharper than the integrating mode image although it took longer to acquire. This difference is illustrated in the corresponding Fourier transforms shown in Fig. 4(b) and (d) where the spots visible in the single event transform are stronger, more numerous and have a lower surrounding noise level. To quantify this difference the MTF enhancement was obtained by comparing the amplitudes of the signal from the strongest spots along the [01] and [10] directions. Similarly, the DQE enhancement was obtained by estimating the noise in the vicinity of a spot and comparing the square of the signal to noise ratios. The results of this analysis and the corresponding results expected from Fig. 3 are given in Fig. 5. The results from the two alternative ways of measuring the improvement in MTF and DQE agree well.

## 4 Discussion and conclusions

When operating in electron counting mode the DQE performance at 120 keV presented in Fig. 3(b) exceeds that of all currently available CCD detectors, the Medpix2 detector and film [8]. The advantage obtained using the counting mode can be expected to be even greater at higher incident energies where the decrease in the ratio of the average to the corresponding variability, in the signal deposited in a thin sensitive layer leads to a lower DQE(0) for integrating mode detectors. The increased range over which electrons backscatter at higher energies also makes it feasible to attempt to disentangle the contributions from primary and backscattered events. Doing so would enable a further improvement in both the higher spatial frequency MTF and DQE.

Even with the improved performance available through using the electron counting mode, any practical detector for use in low dose applications such as single particle cryoEM or cryo-tomography will require a larger field of view than is available with the Vanilla sensor used here. While it is possible to compensate for sample drift during image acquisition, higher frame rates would be advantageous in reducing the overall time for an exposure. Higher frame rates would also increase the apparent rad-hardness of a detector due to the reduction of the integrated leakage current contribution in an individual frame. On the other hand, longer exposure times enforce a welcome increase in spatial coherence and corresponding improved microscope envelope function.

The Vanilla detector does not employ a radhard design and, on top of the increase in leakage current, the exposure to the direct beam results in transient hot pixels. In particular, while the use of a hard reset minimises any image lag, occasionally there is a noticeable long lasting response in a pixel after an event. As this can be above the threshold it will lead to erroneously recording duplicated events. Fortunately such events are typically restricted to a single pixel and can be distinguished from true events, such as those illustrated in Fig. 1, which are generally spread into neighbouring pixels and are absent in the following frames. A practical event counting detector should however incorporate radhard design [15,7] so as to minimise radiation damage and need for extra event processing.

In this paper we have shown that it is possible to obtain both higher MTF and DQE performance from a MAPS detector by operating it in an electron counting mode. We show that using renormalised single event images as an effective probability distribution for the incident electrons arrival position provides an efficient and effective method for processing single electron events. This method, as opposed to choosing the position of the maximum signal or centroid of an event, leads to an improvement in the DQE over all spatial frequencies. We believe that, despite the more than ~5000× increase in raw data, the improvement in imaging performance demonstrated here will make collecting electron event counting images the preferred method with which to obtain low dose images of radiation sensitive samples.

## References

[1] Dainty J., Shaw R. (1974). Image Science.

[2] Meyer R.R., Kirkland A. (1998). Ultramicroscopy.

[3] Mooney P. (2007). Methods Cell Biol..

[4] Faruqi A., Henderson R., Pryddetch M., Turchetta R., Allport P., Evans A. (2005). Nucl. Instrum. Methods A.

[5] Deptuch G., Besson A., Rehak P., Szelezniak M., Wall J., Winter M., Zhu Y. (2007). Ultramicroscopy.

[6] Jin L., Milazzo A.-C., Kleinfelder S., Li S., Leblanc P., Duttweiler F., Bouwer J.C., Peltier S.T., Ellisman M.H., Xuong N.-H. (2008). J. Struct. Biol..

[7] Battaglia M., Contarato D., Denes P., Doering D., Giubilato P., Kim T.S., Mattiazzo S., Radmilovic V., Zalusky S. (2009). Nucl. Instrum. Methods A.

[8] McMullan G., Chen S., Henderson R., Faruqi A. (2009). Ultramicroscopy.

[9] Faruqi A.R., Henderson R., Holmes J. (2006). Nucl. Instrum. Methods A.

[10] Ohta J. (2007). Smart CMOS Image Sensors and Applications.

[11] Allinson N. (2009). Nucl. Instrum. Methods A.

[12] Bohndiek S.E., Arvanitis C.D., Royle G.J., Speller R.D., Clark A.T., Crooks J.P., Prydderch M.L., Turchetta R., Blue A., O’Shea V. (2007). Opt. Eng..

[13] Medipix2 collaboration, URL 〈http://medipix.web.cern.ch/MEDIPIX〉.

[14] McMullan G., Cattermole D., Chen S., Henderson R., Llopart X., Summerfield C., Tlustos L., Faruqi A.R. (2007). Ultramicroscopy.

[15] Eid E.-S., Chan T., Fossum E., Tsai R., Spagnuolo R., Deily J., Byers W.B., Peden J. (2001). IEEE Trans. Nucl. Sci..

